# Fecal microbiota transplantation reverses insulin resistance in type 2 diabetes: A randomized, controlled, prospective study

**DOI:** 10.3389/fcimb.2022.1089991

**Published:** 2023-01-04

**Authors:** Zezhen Wu, Bangzhou Zhang, Fengwu Chen, Rongmu Xia, Dan Zhu, Baolong Chen, Aiqiang Lin, Chuyan Zheng, Ducheng Hou, Xiaoyu Li, Shuo Zhang, Yongsong Chen, Kaijian Hou

**Affiliations:** ^1^ Department of Endocrine and Metabolic Diseases, The First Affiliated Hospital of Shantou University Medical College, Shantou, China; ^2^ Department of Endocrine and Metabolic Diseases, Longhu Hospital, Shantou, China; ^3^ Graduate School of Shantou University Medical College, Shantou, Guangdong, China; ^4^ School of Pharmacy, Fujian University of Traditional Chinese Medicine, Fuzhou, China; ^5^ Clinical Research Center, The Second Affiliated Hospital of Fujian University of Traditional Chinese Medicine, Fuzhou, China; ^6^ School of Public Health, Shantou University, Shantou, China

**Keywords:** fecal microbiota transplantation, type 2 diabetes mellites, metformin, metagenomics, microbiota colonization

## Abstract

**Objectives:**

Recent studies have shown that fecal microbiota transplantation (FMT) improved the metabolic profiles of patients with type 2 diabetes mellitus (T2DM), yet the effectiveness in reversing insulin resistance and increasing metformin sensitivity in T2DM patients have not been reported. In this study, we evaluated the improvements of T2DM patients and their gut microbiota by FMT alone and FMT plus metformin.

**Methods:**

A total of 31 patients with newly diagnosed T2DM were randomized to intervention by metformin, FMT, or FMT plus metformin in the study. Patients were followed up at baseline and week 4 after treatment. Blood and stool samples were collected and subject to analyze clinical parameters and microbial communities by metagenomic sequencing, respectively.

**Results:**

FMT alone and FMT plus metformin significantly improved the clinical indicators HOMA-IR and BMI in T2DM, besides fasting blood glucose, postprandial blood glucose, and hemoglobin A1c that were also controlled by metformin. Donor microbiota effectively colonized in T2DM with slightly higher colonization ration in FMT than FMT plus metformin within 4 weeks, resulting in increased microbial diversity and community changes from baseline after treatment. A total of 227 species and 441 species were significantly alerted after FMT and FMT plus metformin, respectively. FMT were significantly associated with the clinical parameters. Among them, *Chlorobium phaeovibrioides, Bifidibacterium adolescentis* and *Synechococcus* sp.*WH8103* were potential due to their significantly negative correlations with HOMA-IR.

**Conclusions:**

FMT with or without metformin significantly improve insulin resistance and body mass index and gut microbial communities of T2DM patients by colonization of donor-derived microbiota.

## 1 Introduction

Type 2 diabetes mellitus (T2DM) is a metabolic disease characterized by a decrease in pancreatic β-cell mass and function, and represents a failure to compensate for the high insulin demand of homeostatic model assessment of insulin resistant (HOMA-IR) states (Aguayo-Mazzucato et al., 2019). The occurrence of HOMA-IR is a key predictor of the development of T2DM (Wallace et al., 2019). The global prevalence of T2DM is alarmingly high with an estimated population of 370 million, which is predicted to be doubled by 2030 (Wild et al., 2004). This dramatic increase in T2DM poses an immense public health crisis and medical challenge. Recent research showed that intestinal dysbiosis is a key factor in the development of metabolic endotoxemia and T2DM (Zhao et al., 2018; Thingholm et al., 2019; Wu et al., 2022).

The human intestines harbor a complex community of intestinal bacteria (Skelly et al., 2019), viruses (Ingle et al., 2019), fungi (Li et al., 2019), and protists (Chudnovskiy et al., 2016). Recent data confirmed that intestinal dysbiosis was associated with the development of metabolic syndrome, especially T2DM (Karlsson et al., 2013; Que et al., 2021; Hou et al., 2022). The composition and quantity of intestinal microbiota in diabetic patients have been found to differ from healthy individuals (Marchesi et al., 2016; Chen et al., 2019). Current studies showed that intestinal microbiota is involved in the development of obesity and insulin resistance in diabetes mellitus *via* different mechanisms, and many hypoglycemic drugs result in changes of intestinal microbiota (Su et al., 2015; Li et al., 2017). Metformin is now widely used in T2DM treatment, and recent evidence suggests that the intestinal microbiota serves as a metformin action site (Pollak, 2017; Rodriguez et al., 2018; Foretz et al., 2019). Sun et al. indicated that metformin acted partially *via* a B. fragilis-glycoursodeoxycholic acid (GUDCA)–intestinal farnesoid-X receptor (FXR) axis to improve metabolic dysfunction (Sun et al., 2018). The therapeutic potential of fecal microbiota transplantation (FMT) in diabetes has been discussed in many papers (Wang et al., 2019; Aron-Wisnewsky et al., 2019; Ng et al., 2022; Hou et al., 2022). For example, Groot et al. revealed that FMT could halt the decline in endogenous insulin production and featured intestinal microbiota were linked to remaining beta cell function of type 1 diabetes (T1DM) patients (de Groot et al., 2021). Siew et al. reported that repeated FMTs enhance the level and duration of microbiota engraftment in obese patients with T2DM (Ng et al., 2022). However, no study has reported the application of FMT in assisting the efficacy of metformin in T2DM treatment. Thus the aim of our work was to evaluate adjunctive FMT with metformin in southeast Chinese population with T2DM.

We proposed that FMT would alter T2DM patients’ microbial ecology and thereafter improve the blood glucose and insulin sensitivity. An FMT clinical trial for the intervention of T2DM patients with metformin, FMT alone, and FMT plus metformin was initiated. The primary outcome was the evaluation of changes in insulin sensitivity (HOMA-IR and HOMA-HBCI), postprandial blood glucose (PBG), fasting blood glucose (FBG), hemoglobin A1c (HbA1c), and BMI between baseline and after 4 weeks of intervention. The secondary outcomes were the proportion of subjects acquiring least 20% of microbiota from the donor after FMT at week 4.

## 2 Materials and methods

### 2.1 Study population

We recruited 29 adult T2DM patients, following the diagnostic criteria of the American Diabetes Association (ADA) for T2DM in 2019. We obtained written informed consent from all patients before screening. All patients volunteered to participate in the trial and exhibited good compliance and did not replace diabetic drugs in the study cycle. Patients were excluded if they had other diagnoses: 1) acute and chronic infectious diseases, gastrointestinal diseases, severe heart insufficiency, severe liver and kidney insufficiency, and/or other diseases or complications; 2) other gastrointestinal diseases that may affect drug absorption; 3) pregnant and lactating women; 4) people with allergies; 5) patients who have used other hormone therapy within the past three months; 6)Leukopenia or abnormal granulocytes; 7) cardiovascular and cerebrovascular diseases that first occurred in the past three months; 8) Participants in other clinical trials during the same period; 9) a history of human immunodeficiency virus (HIV) seropositivity after laboratory screening; and 10) hepatitis B virus surface antigen (HBsAg) positive or hepatitis C virus antibody (HCV-Ab) history after laboratory screening. During this period, the research team instructed all participants to maintain their original eating habits before and after the intervention, including total calories, types, diet culture, etc., and to maintain light to moderate physical activity (the same intensity) and avoid heavy physical activity. The study has been approved by the Longhu Hospital, The First Affiliated Hospital of Shantou University Medical College Ethics Committee in Shantou, China(Ethics number:LHLL2019001), and was registered at Chinses Clinical Trial Registry.(Registration number: ChiCTR1900024636).(http://www.chictr.org.cn/showprojen.aspx?proj=41166).

### 2.2 Research plan and outcomes

This study used FMT as an auxiliary method to compare the therapeutic effects of solely metformin, solely FMT and FMT combined with metformin onT2DM patients. Eight T2DM patients received FMT plus metformin treatment, 9 patients underwent FMT alone and 12 patients received solely metformin treatment. The primary research objective was to evaluate the efficacy of FMT in assisting the metformin treatment in T2DM adult patients from the aspects of blood sugar control and insulin resistance. The secondary research objective was to observe the influence of FMT on the bacterial engraftment from donor microbiota during baseline inspection and week 4 intervention. We classified microbiota species identified in the recipients into four types and mainly focused on the donor-associated species as previously defined (Ng et al., 2022).

### 2.3 Intervention procedures

Screening of study donors was based on previous reports (Wu et al., 2020; He et al., 2021). The gut microbiota of ten qualified-donors was isolated automatically using the fecal microbiota extractor TG-01 (Treat-gut company, Guangzhou, China). The procedures involved mixing of stool with saline solution and multiple filtrations with different pore sizes, which were completed in Xiamen Treat-gut Biotechnology Co. ltd. We studied the effects of FMT *via* nasojejunal feeding tubes on clinical phenotypes and intestinal microbiota before and 4 weeks after intervention. The interventions consisted of metformin, FMT alone, and FMT plus metformin. For the FMT, before transplantation, patients had to be confirmed their stomach remained empty for more than 4 hours, then 200 mL of FMT solution containing 50g bacterial sludge was injected *via* the nasointestinal tube to the anterior jejunum. The position was confirmed by X-ray. Two hours after FMT, the participants were allowed to have a small amount of liquid diet. Blood and stool samples of all participants were collected at baseline (week 0) and week 4 after intervention for biochemical and microbiota assessments.

### 2.4 Fecal microbiota analysis by metagenomic sequencing

Fecal samples from donor and T2DM patients were collected on the day of the medical examination and immediately frozen at −80°C. Fecal genomic DNA was extracted using the QIAamp Fast DNA Stool Mini Kit (Qiagen, CA, USA). DNA samples were stored at −20°C before use as templates for next-generation sequencing library preparation. Samples were fragmented to an average insert size of 400 bp and sequenced by Illumina Nova seq with PE 150 reagents. Reads were trimmed using KneadData with default parameters to filter the sequencing adapter, low-quality reads, and the human genome. The taxonomic composition was processed using kraken2 (Erem et al., 2014) with default parameters.

### 2.5 Statistical analysis

Microbiota alpha diversity Shannon and Chao1 were calculated using the R program package ‘vegan’ (version 2.5.6). β-diversity metrics were obtained with rda and PERMANOVA with the adonis function. Principal Components analysis (PCA) was performed using the package vegan. Different analysis was performed to identify taxa with differentiating abundance in the different groups (Krentz and Bailey, 2005). The corr.test function was used to analyze the correlation between the microbial taxa and clinical indexes. Statistical significance was taken as p value <0.05.

## 3 Results

### 3.1 Characteristics of the study population

**Figure 1:**
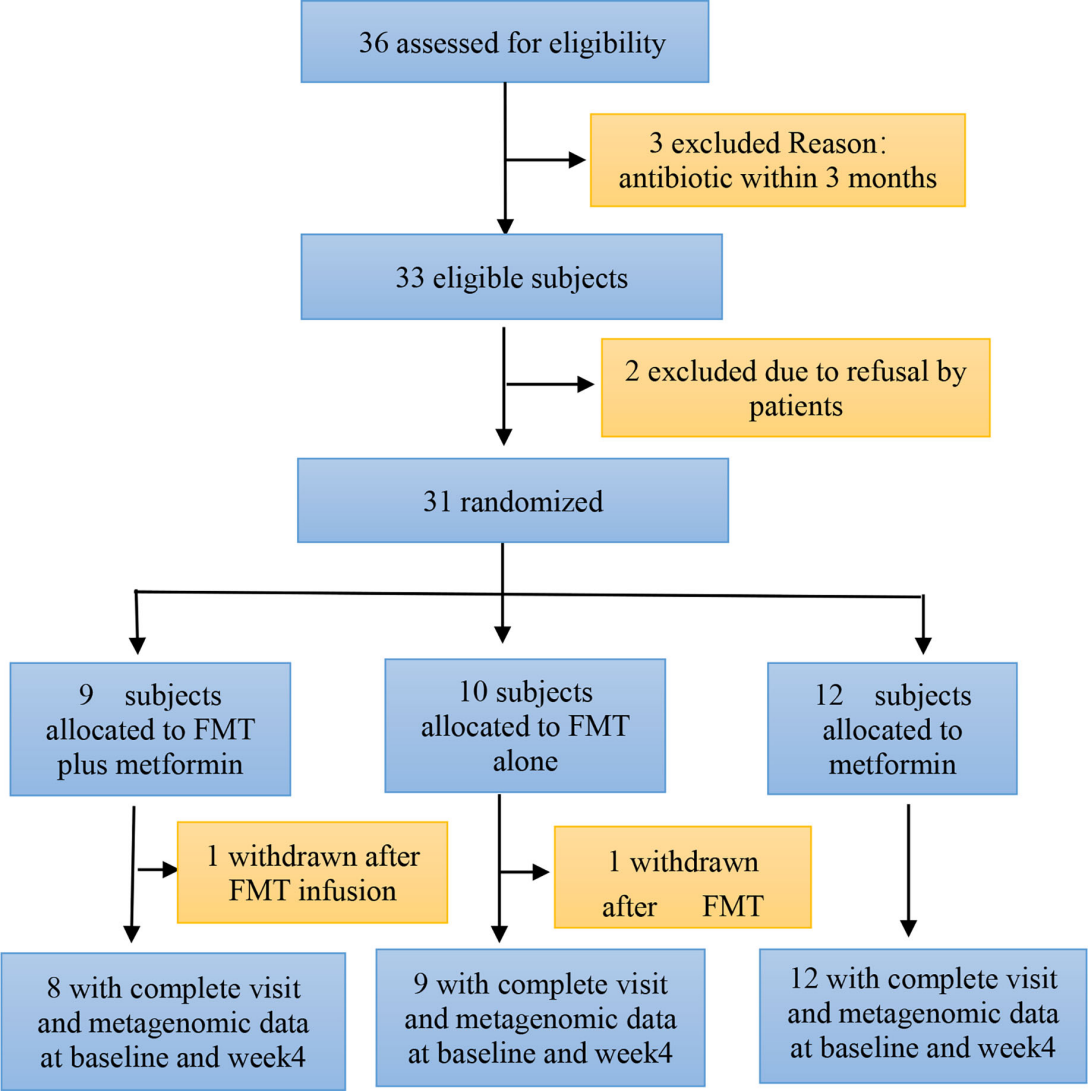
Consort diagram of the study flow. FMT, faecal microbiota transplantation.

A total of 36 patients with T2DM were assessed for eligibility, of whom 31 were recruited and randomized to either FMT plus metformin, FMT alone, or metformin from July 2019 to Oct 2021. One of participants in both FMT plus metformin and FMT alone group withdraw after FMT infusion. Finally, 29 patients allocated to FMT plus metformin (n=8), FMT alone (n=9), or metformin (n=12) completed their follow-up assessment at both baseline and week 4 (**Figure 1**). Demographic characteristics of patients in the three groups were comparable. Males constituted 48.3% of the patients (n=14), and the median BMIs for FMT plus metformin, FMT alone, and the metformin groups were 27.46, 27.29, and 27.01 kg/m^2^, respectively. Ten healthy donors (90% male, median BMI: 21.56kg/m^2^) provided stool for the FMT solution.

### 3.2 Blood glucose, insulin resistance, and BMI improvement after intervention

**Figure 2:**
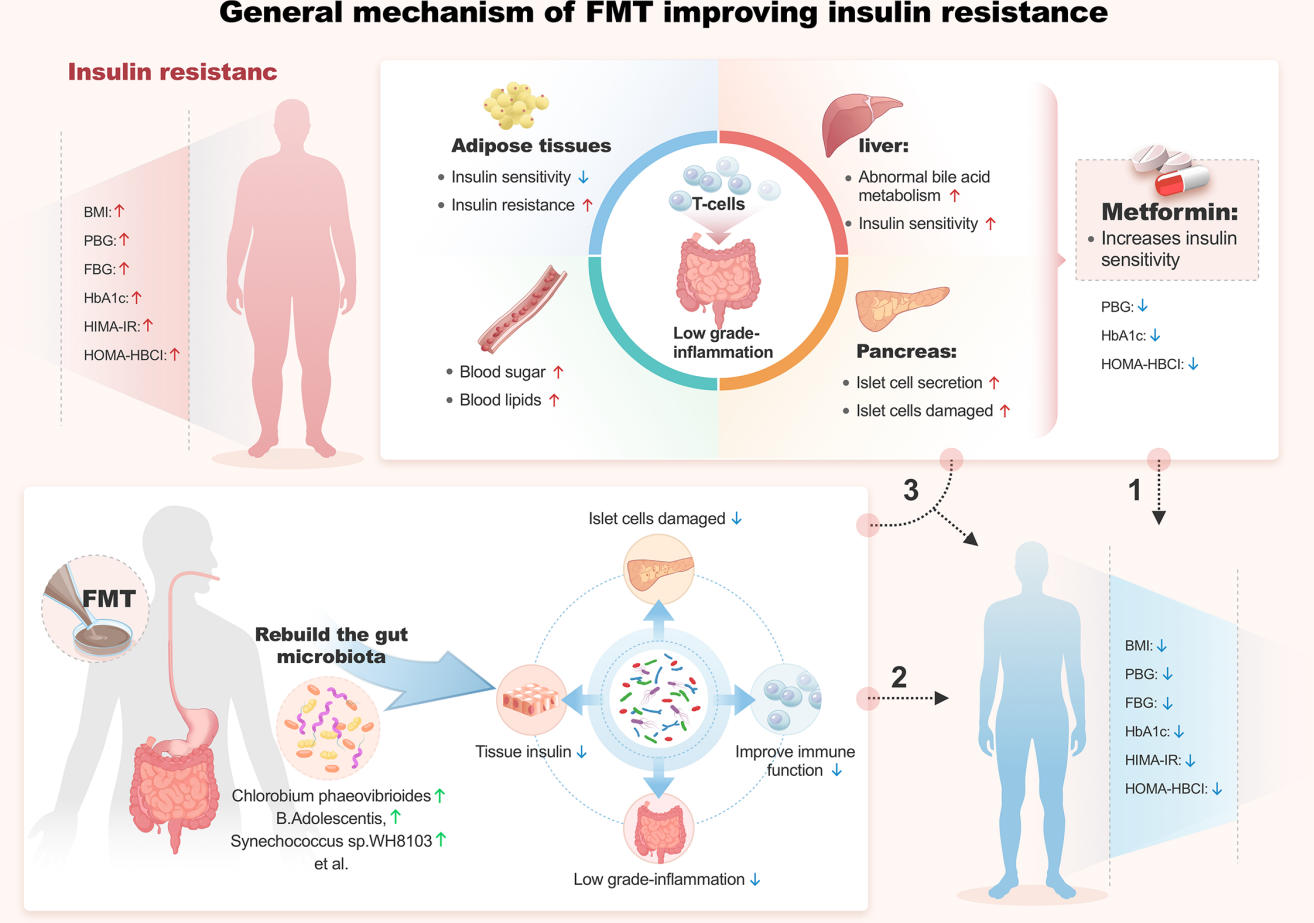
General mechanism of FMT improving insulin resistance.

Participants in the three treatment groups had significant (P<0.05) improvement in fasting blood glucose (FBG); postprandial blood glucose (PBG), hemoglobin A1c (HbA1c), and HOMA-HBCI at week 4 after intervention compared with the baseline (**Table 1**). More importantly, patients in FMT alone and FMT plus metformin had significantly decreased HOMA-IR (p<0.05) and BMI (p<0.05) at week 4 after FMT intervention, while no differences were observed for those in metformin group (**Table 1**). Significantly lower UA, TG, and Globulin were observed in FMT plus metformin group (**Figure 2**).

**Table 1:** Clinical data included in this study.

	Metformin (n=12)			FMT (n=9)			FMT plus metformin (n=8)		
Index	Week 0	Week 4	*p* value	Week 0	Week 4	*p* value	Week 0	Week 4	*p* value
FBG	9.08±2.02	8.37±1.98	**0**	14.3±1.99	8.12±0.72	**0**	9.24±1.92	6.78±1.68	**0.01**
PBG	13.96±2.61	11.35±1.92	**0**	18.48±5.59	11.34±2.2	**0**	14.21±4.77	9.08±2	**0.01**
HbA1c	8.56±1.13	8.29±1.09	**0**	10.75±1.85	8.57±1.97	**0.01**	9.1±2.14	8.76±2.21	**0.01**
HOMA-HBCI	41.55±20.25	58.04±37.21	**0.03**	22.9±16.06	44.65±23.03	**0**	41.86±19.77	86.47±109.22	**0.01**
BMI	27.51±0.89	27.42±0.99	0.2	27.2±0.95	26.37±0.87	**0**	27.27±1.07	26.46±1.28	**0.01**
HOMA-IR	3.99±1.39	4.51±2.03	0.52	6.73±2.88	3.55±1.58	**0**	5.57±5.91	3.61±4.15	**0.01**
PCP	4.58±2.09	4±1.26	0.13	3.85±2.19	4.5±2.35	0.13	3.86±3.21	4.62±4.21	0.84
FINS	10.08±3.03	12.21±5.34	0.27	11.05±5.25	9.93±4.63	0.07	12.33±9.65	12.04±14.81	0.25
AFU	30.32±4.02	28.49±3.43	0.09	25.88±5.75	24.5±4.24	0.25	19.69±7.16	24.23±7.58	**0.03**
FCP	1.89±1.12	1.87±0.72	0.62	1.57±0.99	1.76±0.71	0.43	1.68±1.22	1.99±0.93	0.74
Creatinine	87.67±94.69	75±39.53	0.4	64.67±11.07	68±12.45	0.11	66.38±12.39	72.25±14.66	0.09
GGT	30.93±13.1	33.6±16.84	0.73	39.03±56.67	35.1±51.57	0.36	31.27±17.9	23.32±12.02	0.15
D-BIL	4.21±1.09	3.61±1.45	0.08	3.96±1.37	3.66±1.04	0.43	3.12±1.43	3.7±1.24	0.53
ALP	83.75±15.79	67.33±11.24	**0**	73.22±29.39	69.89±16.71	1	68.88±11.64	67.12±14.19	0.36
TBA	6.82±2.31	3.36±2.24	**0**	6.28±7.42	3.53±1.68	0.31	3.99±2.68	4.22±3.42	0.95
TBIL	11.4±2.77	11.08±4.37	0.62	12.71±4.1	12.19±3.81	0.82	13.04±7.3	13.26±4.71	0.95
APO-B	0.86±0.24	0.97±0.18	0.08	1.03±0.35	1.11±0.26	0.65	0.84±0.21	0.83±0.21	0.74
PINS	37.38±22.24	31.74±14.88	0.2	35.72±24.36	35.23±28.05	0.82	31.31±21.07	14.3±14.47	0.04
LDL-C	2.96±1.09	3.12±0.74	0.57	2.98±0.56	3.52±0.79	0.16	2.78±1.12	2.74±0.75	0.74
BUN	5.22±2.46	5.77±2.41	0.13	5.21±0.81	5.64±1.2	0.25	5.68±1.26	5.01±2.1	0.55
ALT	30.23±34.83	35.03±46.76	0.46	22.8±11.11	21.43±5.86	0.73	29.55±19.71	24.2±6.66	0.38
HDL-C	1.19±0.24	1.25±0.27	0.14	1.13±0.28	1.16±0.2	0.64	1.32±0.52	1.27±0.46	0.2
CHE	9196.42±1539.21	9511.5±1510.36	0.47	9830.11±2714.23	9413.89±1709.88	0.73	6051.88±3700.28	6019.25±3762.15	0.74
UA	366.42±81.33	374.5±67.24	0.73	4061.56±126.12	366.11±74.71	0.57	377.38±59.76	360.12±60.41	**0.01**
TG	1.74±0.57	1.52±0.46	0.23	3.84±5.5	2.3±1.36	0.57	2.18±1.44	1.26±0.29	**0.04**
TC	4.72±1.35	4.87±0.79	0.64	5.37±1.02	5.48±0.36	0.73	4.8±1.13	4.44±0.71	0.64
AST/ALT	1.17±0.38	0.86±0.24	**0.01**	1±0.31	1.02±0.23	0.83	1.12±0.6	1.06±0.24	0.94
TP	75.22±5.45	72.91±4.03	0.11	73.51±10.05	74.67±3.6	0.57	72.36±11.66	68.65±6.16	0.31
MAO	4.25±0.97	4.25±1.14	1	6.67±6.86	4.44±2.3	1	5.55±1.44	7.06±2.48	0.14
I-BIL	6.8±1.87	7.27±2.96	0.62	8.76±2.91	8.94±2.34	1	9.92±6.87	9.03±4.17	0.64
Globulin	26.82±3.85	26.95±4	0.97	26.37±6.58	27.88±3.26	0.43	27.89±6.11	23.92±3.38	**0.04**
AST	27.88±13.51	24.23±17.34	**0.03**	20.29±6.06	21.59±5.57	0.91	25.24±7.82	22.88±6.27	0.23
APO-A	1.28±0.21	1.23±0.16	0.24	1.43±0.29	1.35±0.15	0.82	1.4±0.19	1.31±0.41	0.38
Albumin	48.44±2.89	46.36±1.99	**0.02**	47.04±3.77	47.3±2.48	0.91	44.47±6.43	44.86±5.87	0.89

The data are shown as the mean±SD. N=12 in the metformin group, N=9 in the FMT group and n=8 in the FMT plus metformin group for all outcomes. FMT, fecal microbiota transplantation; FBG, fasting blood glucose; PBG, postprandial blood glucose; HbA1c, hemoglobin A1c.

HOMA-HBCI=20×FINS/(FBG-3.5); BMI, body mass index; HOMA-IR=(FBG×FINS)/22.5; PCP, postprandial c-peptide; FINS, fasting insulin; AFU, α-L-fucosidase; FCP, fasting c-peptide; GGT, γ-glutamyl transpeptadase; D-BIL, direct bilirubin; ALP, alkaline phosphatase; TBA, total bile acid; TBIL, total bilirubin; APO-B, apolipoprotein B; PINS, postprandial insulin; LDL, low-density lipoprotein; BUN, urea nitrogen; ALT, alanine aminotransferase; HDLC, high-density lipoprotein; CHE, cholinesterase;UA, uric acid; TG, triglyceride; TC, total cholesterol;TP, total protein; MAO, monoamine oxidase; I-BIL, indirect bilirubin; AST, aspartate aminotransferase; APOA, apolipoprotein A. Bold values represents statistically significant indicators.

**Figure 3:**
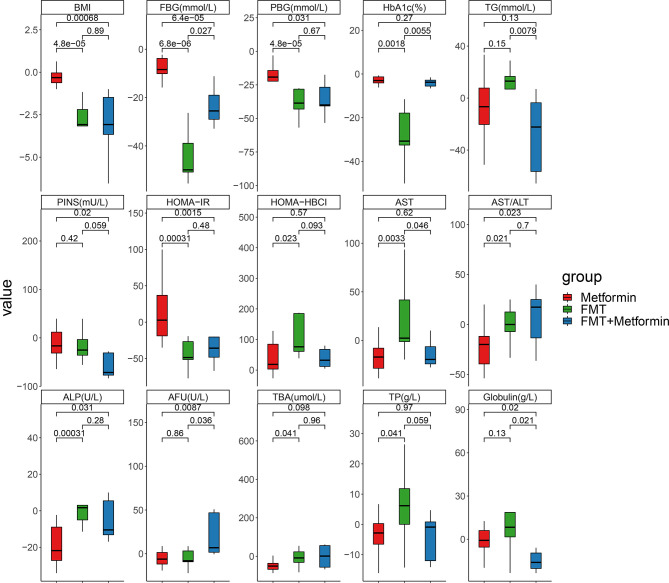
Fold changes of clinical indexes based on week 4 divided by week 0 in T2DM patients with different interventions. Pairwise comparisons between groups were conducted using the Wilcox test. Metformin, n=12 patients; FMT, n=9 patients; FMT plus metformin, n=8 patients. *p*<0.05 is defined as statistically significant.

We further evaluated the magnitude of change among the three groups. Percentages of improvements in PBG, FBG, HOMA-IR, BMI, AST/ALT and ALP were significantly higher in the FMT alone and FMT plus metformin than in metformin group (*p*<0.05) (**Figure 3**). Improvements in HOMA-HBCI, HbA1c, AST, TBA and TP were observed significantly higher in FMT alone than metformin group. Additionally, FBG and HbA1c decreased more in FMT plus metformin than in FMT alone. No significant differences in changes of PBG, PINS, HOMA-IR, HOMR-HBCI, AST/ALT, ALP, TBA, TP and BMI were observed between the two FMT-associated treatments (*p>*0.05) (**Figure 3**).

### 3.3 Microbiota alterations associated with FMT intervention

**Figure 4:**
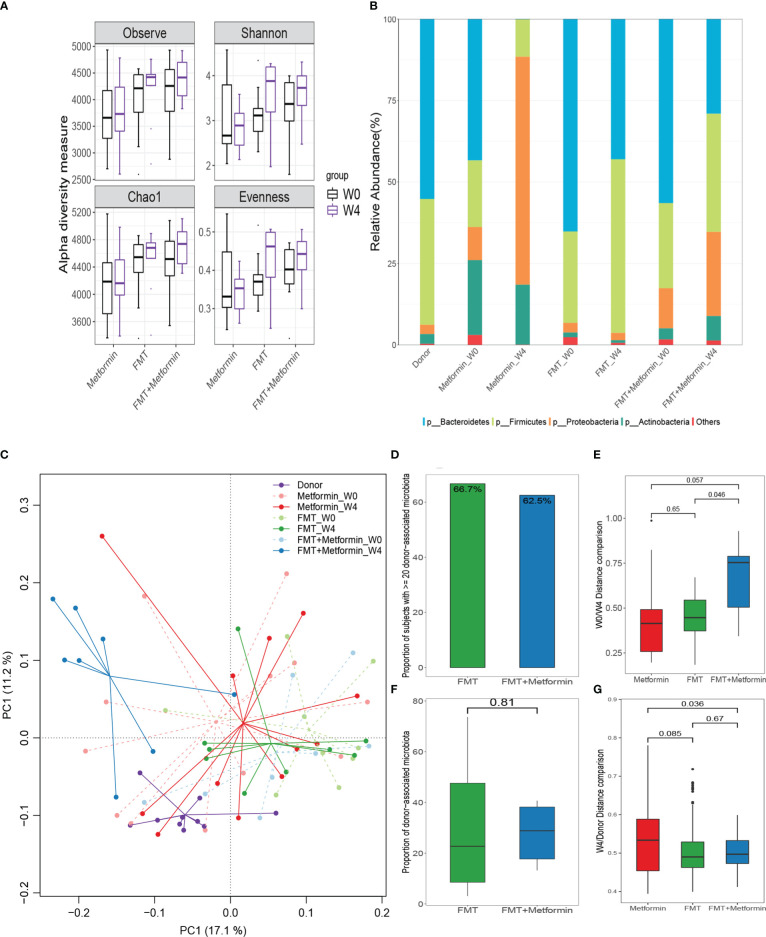
Changes in gut microbiota between week 0 and week 4 in T2DM patients after interventions. **(A)** Differences in alpha diversity indexes, microbial richness (Observed index and Chao1 index), Shannon diversity, and evenness. **(B)** Relative abundances of the top phyla in the these groups; Changes in gut microbiota and microbial colonization of donor-derived microbiota in subjects with type 2 diabetes. **(C)** Differences in beta diversity between baseline (W0) and week 4 (W4)  after intervention by metformin, FMT, or FMT plus metformin, visualized by PCA. **(D, F)** The colonization of donor-derived microbiota in FMT alone and FMT plus metformin groups. **(E)** Changes of Euclidean distance between W0 and W4. **(G)** Changes of Euclidean distance between W4 and donors.

Microbial richness (observed taxa and Chao1) and Shannon diversity were obviously (P < 0.05) improved at week 4 after FMT compared with the baseline, although the significance is marginal. Moreover, the evenness was significantly (P<0.05) increased by FMT in FMT alone group at week 4 (**Figure 4A**). No obvious changes in diversity indexes were observed in metformin group. As expected, Bacteroidetes, Firmicutes, and Proteobacteria were the dominant taxa in donor alone or overall samples (**Figure 4B**). Relative abundance of Bacteroidetes was decreased, while Firmicutes increased after intervention in both FMT alone and FMT plus metformin groups. An uncommon microbial composition was observed at week 4 after metformin treatment, with a surprising high proportion of Proteobacteria and almost absence of Bacteroidetes.

### 3.4 β-diversity and microbial colonization

**Figure 5:**
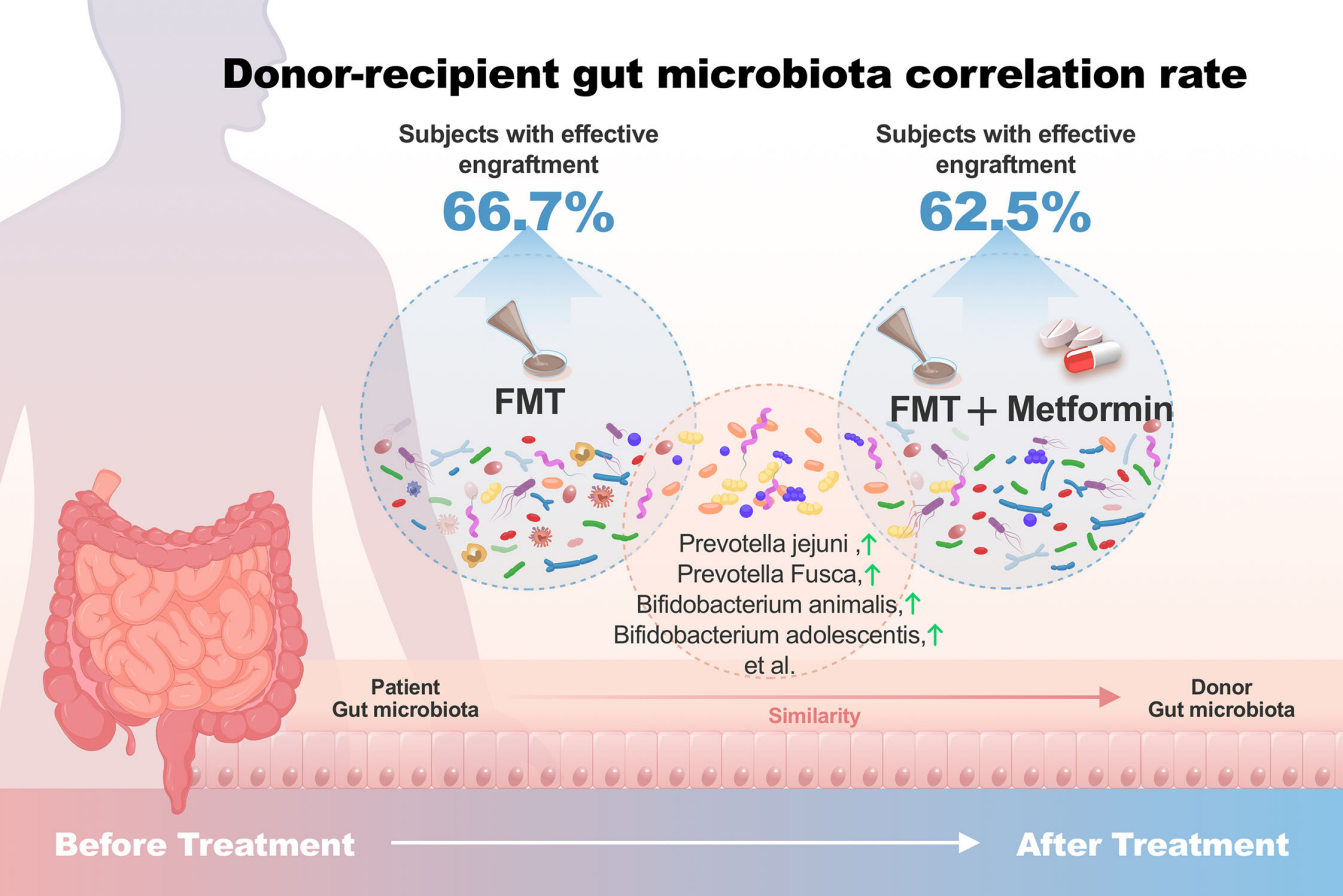
Donor-recipient gut microbiota correlation rate. Subjects with effective engraftment: Percentage of subjects with ≥20% donor-associated microbiota.

The results of β-diversity based on Euclidean distance showed that the intestinal microbiota changed at week 4 compared with week 0 with the three treatments. The gut microbial communities in FMT plus metformin group were significantly different (PERMANOVA, p < 0.05) between week 4 and week 0, while there was no significant difference in FMT alone nor in metformin group (**Figure 4C**). Among the three groups, the metformin group had the smallest change in beta diversity after 4 weeks, followed by the FMT group, and that the FMT plus metformin group had the largest change, although significant difference were observed among the three groups (**Figure 4E**). Similarly, the Euclidean distance between week 4 from each of the three groups and the donor was calculated. The results showed that the distance between the metformin group and the donor was the largest, and the FMT plus metformin group was the smallest, indicating that the gut microbiota in the FMT plus metformin group was similar to that of the donor after treatment (**Figure 4G**, **Figure 5**).

The colonization of donor-derived microbial species was analyzed and detected in post-FMT samples from all recipients in both FMT alone and FMT plus metformin groups, with percentage ranging from 3.1% to 73.7%.The results showed that 6 patients (66.7%) in the FMT group and 5 patients (62.5%) in the FMT plus metformin group achieved ≥20% donor-derived microbial species, which was considered as effective colonization. However, there was no significant difference in colonization rate between the two groups (p > 0.05) (**Figures 4D, F** **)**.

### 3.5 Taxa significantly associated with clinical improvements

**Figure 6:**
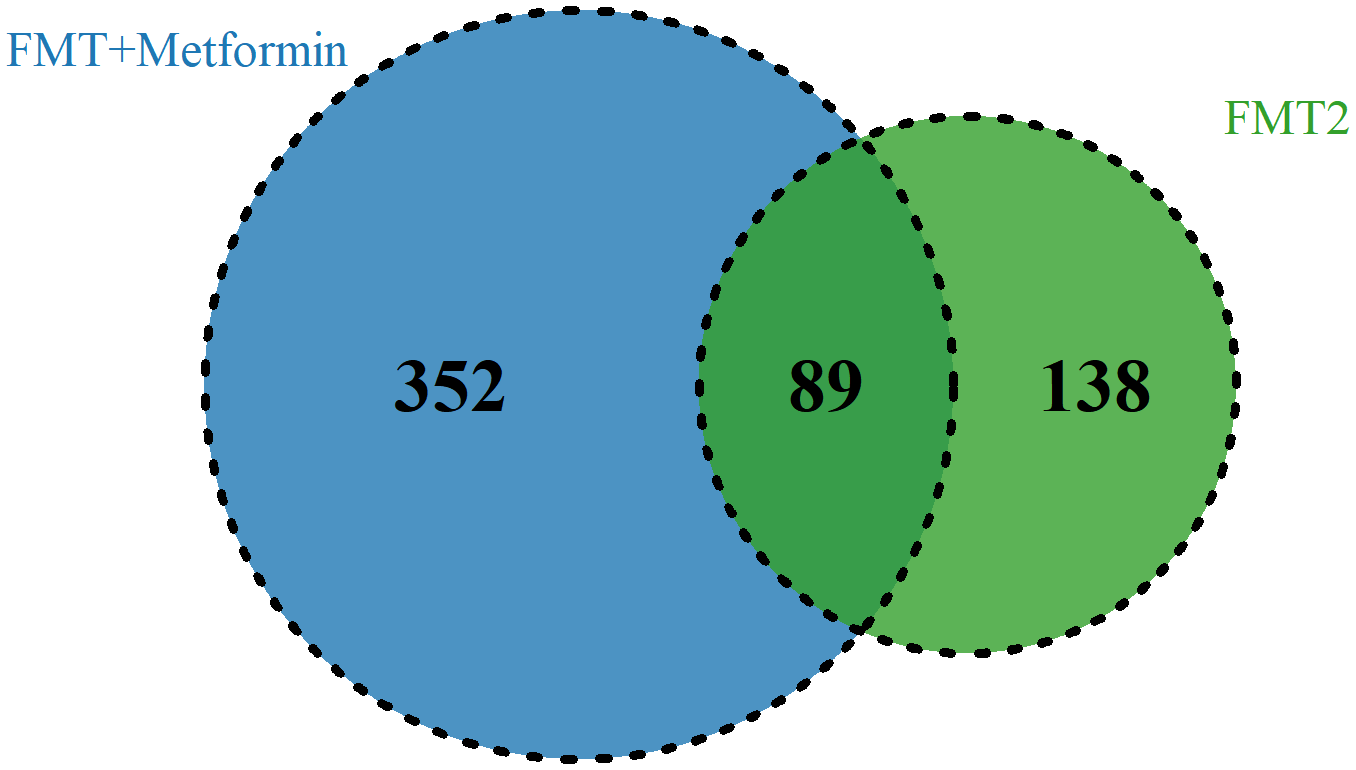
The commonly and significantly different species of between the FMT alone and FMT plus metformin groups.

**Figure 7:**
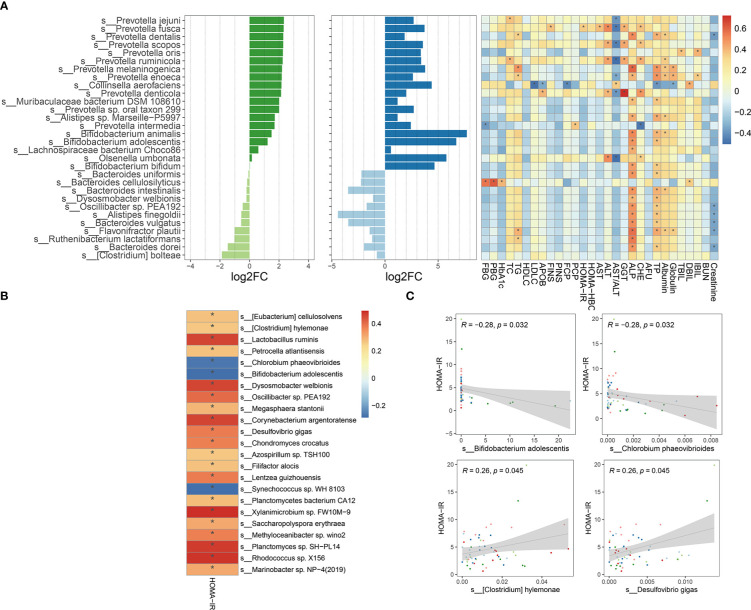
Microbial species significantly changed after intervention and their correlation with clinical indicators. **(A)** The top 30 significantly changed species after FMT or FMT plus metformin and their correlation with clinical indicators based on samples from W0 and W4 of these two groups. **(B)** Significant corrections between HOMA-IR and significantly changed species with relative abundance greater than 0.001%, based on all samples from T2DM patients at W0 and W4 in metformin, FMT alone, and FMT plus metformin group. **(C)** 4 gut microbes significantly related to HOMA-IR. "*" means that P value is < 0.05.

To explore the taxa associated with improvement in clinical efficacy, the differences between baseline and week 4 after intervention in FMT alone and FMT plus metformin groups were analyzed by Wilcoxon-rank sum test. A total of 7 phyla, 57 families, and 133 genera level were significantly (p < 0.05) different after intervention in FMT alone group, while the numbers were 10, 63 and 206 in the FMT plus metformin group (**Table S1**). With cut-off of relative abundance bigger than 0.001%, there were 227 species and 441 species that were significantly different between baseline and week 4 in in FMT alone and FMT plus metformin groups, respectively. Among them, 89 species were shared in these two groups (**Figure 6**). The species with increased relative abundance at week 4 after treatment mainly belonged to the genera *Prevotella* and *Bifidobacterium*, including *Prevotella jejuni*, *Prevotella Fusca*, *Bifidobacterium animalis*, and *Bifidobacterium adolescentis*. Correlation analysis based on the samples from these two groups showed that *Provetella* were positively correlated with ALP and TP, while *Bifidobacterium* were negatively correlated with CHE, FBG, PBG, TC and PINS (**Figure 7A**). Additionally, *Collinella aerofaciens* was strongly negatively correlated with FBG, while PGB was strongly positively correlated with *Clostridium bolteae* and HOMA-IR was strongly positively correlated with *Dysosomobacter Wekbionis* (p < 0.05).

Since improvement in HOMA-IR is urgent in treatment of T2D, we further explored the species by conducting correlation analysis between HOMAR-IR and the species significantly different either after FMT or FMT plus metformin, based on samples from T2DM patients at baseline and week 4 after intervention. The results showed that most of the significantly associated species were positively correlated with HOMA-IR (**Figure 7B**), including *Lactobacillus ruminis*, *D. welbiois* and *Xylanimicrobium* sp. *FW10M-9*, while *Chlorobium phaeovibrioides*, *B. adolescentis* and *Synechococcus* sp.*WH8103* were strongly negatively correlated with HOMA-IR, which were increased at week 4 after intervention by FMT or FMT plus metformin (**Figure 7C**).

## 4 Discussion

This study aimed to evaluate the improvements of T2DM patients and their gut microbiota by FMT alone and FMT plus metformin, compared with metformin. Results showed that FMT alone and FMT plus metformin significantly improved insulin resistance (HOMA-IR), HOMA-HBCI, BMI, FBG, and PBG within 4 weeks after intervention, and modified gut microbial communities by colonization of donor-derived microbiota. Correlation analysis revealed that *B. adolescents*, *C. phaeovibrioides*, and *S.* sp.*WH8103* were significantly negatively correlated with HOMA-IR, the urgent indicator in treatment of T2DM. In short, this study support that FMT alone and FMT plus metformin can improve insulin resistance and other indicators of patients T2DM by colonizing donor-derived microbes and modifying gut microbiota in diversity and specific species.

Metformin is currently widely used as the first-line drug for the treatment of T2DM patients, as recommended by clinical guidelines, due to the improved glycemic profile and reduction in cardiovascular mortality without the risk of hypoglycemia and/or body weight gains (Buse et al., 2016; Foretz et al., 2019). Previous research showed that metformin decreased BMI, HbA1c, and FBG during an initial 4-month study period (Shin et al., 2014). In another 52 week study, newly diagnosed T2DM patients who used metformin for blood glucose intervention had significantly lower BMI, FPG, PPG and HbA1c after treatment compared with baseline in the MET group (DeFronzo et al., 2016). The present study also observed the improvements in HbA1c and FBG of T2DM after intervention of metformin, but not BMI and other indicators including HOMA-IR, which was improved by FMT alone and FMT plus metformin. This may be related to FMT peripheral insulin sensitivity and other mechanisms to improve blood glucose control, while MET mainly plays a role in the liver by reducing liver glucose output (Wu et al., 2017). More and more studies have shown that the process of metformin’s role has a certain relationship with gut microbiota. Intravenous metformin does not improve blood sugar (de la Cuesta-Zuluaga et al., 2017). However, the level of metformin in the intestine is 100-300 times higher than that in the serum, making the intestine the main reservoir of dimethyl lenediamine for human (Carvalho and Saad, 2013; Duparc et al., 2017; Depommier et al., 2020). In addition, metformin can also change the composition of gut microbiota by increasing *Akkermansiamuciniphila*, a microbiota that stimulates SCFA production (short chain fatty acids), which is degraded by mucin (Ma et al., 2019). The production and regulation of SCFA is considered to be one of the mechanisms of probiotics to promote health results (Hartstra et al., 2015). In our experiment, the FBG and HbA1c in the FMT alone group decreased more than those in the FMT plus metformin group, which may be related to the improvement of microbial population structure promoted by metformin.

The patients included in our cohort were diagnosed with T2DM and were not receiving prior regular drug treatment or dietary intervention. These T2DM patients had poorly controlled blood glucose or serious insulin resistance and received no other medications for the treatment of other diseases. During the observation period, the enrolled patients did not perform any physical exercise other than daily life activities and received generally consistent dietary regulation. This design enabled us to diminish the effects of major confounding factors that have a known impact on the gut microbiome. Clinically, not all T2DM patients benefit from the use of metformin or respond to metformin quickly. For example, some patients exhibit strong insulin resistance and weak insulin secretion function despite metformin treatment. Therefore, FMT was used to assist metformin treatment in these patients to quickly improve their sensitivity to metformin.

The present study demonstrated that, although metformin could improve the blood glucose, the addition of FMT promoted the improvement further. Many studies have shown that gut microbiota are closely related to glucose metabolism (Musso et al., 2011), insulin resistance (Lee et al., 2019), and insulin secretion (Kootte et al., 2017). The mounting evidence of a causal role of the gut microbiota in T2DM has led to the development of targeted therapeutic approaches that are designed to alter the microbial composition (Vrieze et al., 2012; Rinott et al., 2021). Fecal microflora transplantation (FMT) is a method to treat diseases by rebuilding gut microbiota (Wu et al., 2011). FMT has consistently demonstrated a capability to overcome dysbiosis *via* a profound sustained effect on the gut microbiome, which may become a new way to treat T2DM (Belenguer et al., 2006). Previous experiments using FMT to interfere with metabolic syndrome showed that FMT improved insulin sensitivity, increased gut microbial diversity, and significantly increased butyric acid producing bacteria in patients with metabolic syndrome (Tolhurst et al., 2012; Yadav et al., 2013). Our results are consistent with previous findings on transient lean donor -9gut microbiota in patients with metabolic syndrome, which showed a significant improvement in peripheral insulin sensitivity at 6 weeks (Belenguer et al., 2006). The species with increased relative abundance 4 weeks after FMT mainly belong to *ruminis, D. welbiois* and *Xylanimicrobium* sp. *FW10M-9* were significantly negatively correlated with HOMA-IR, while *Chlorobium phaeovibrioides, B. adolescentis* and *Synechococcus* sp.*WH8103* were strongly negatively correlated with HOMA-IR. Previous studies have shown that long-term intake of fat rich diet is related to the increase of *Bacteroids*, and *vegetarians* are conducive to the proliferation of *Proctor bacilli. Proburella* was also found to be associated with improved glucose tolerance induced by dietary fiber (Su et al., 2022). Bifidobacterium, on the other hand, produces acetate and lactate during carbohydrate fermentation, which are converted into butyric acid by other gut microbiota (Li et al., 2016). Among them, butyric acid plays an important role in regulating insulin secretion (Turnbaugh et al., 2009; Giongo et al., 2011). Our results again show that *Plasmobacterium* and *bifidobacterium* may be the key organisms related to T2DM improvement, which is consistent with the previous research of others (Aggarwala et al., 2021).

A decent engraftment of donor-associated microbiota taxa in recipients during the FMT procedure is one of the prerequisites that guarantees the efficacy of FMT. It is generally believed that the diversity of gut microbiota is closely related to gut health, and the colonization rate of donor flora in the recipient is an important indicator to evaluate the success rate of transplantation. In our study, about 2/3 of the FMT group and the FMT plus metformin group reached the target of ≥ 20% donor derived microbial species, which was significantly increased by 23 compared with other previous studies (Herfarth et al., 2019). This is considered to be effective colonization, which is closely related to our strict donor selection before transplantation. Previous studies have shown that the number of Firmicutes in diabetes patients is lower than that in non-diabetes patients (Mocanu et al., 2021). The progression of diabetes is associated with a decrease in the number of Firmicutes and Bacteroidetes (Mayo and Sinderen, 2010). Firmicutes usually participate in the transport of nutrients and promote the absorption and fermentation of SCFA in indigestible carbohydrates (Pinzone et al., 2012). In our clinical experiment, at the 4th week after FMT, Microbial richness (observed taxa and Chao1) and Shannon diversity were significantly improved compared with the baseline. After FMT and FMT plus 8 metformin groups, the relative abundance of Bacteroidetes decreased, while Firmicutes increased, which indirectly proved that the health of receptor gut microecology was improved after FMT. Aggarwala et al. observed that bacterial strain engraftment in Clostridium difficile infection (CDI) recipients independently explained (precision 100%, recall 95%) the clinical outcomes (relapse or success) after initial and repeat FMT (Pinzone et al., 2012). Low donor FMT engraftment resulted in low clinical efficacy of FMT in patients with Antibiotic-dependent pouchitis (ADP) (Bordalo Tonucci et al., 2017). Siew et al. demonstrated that FMT repeated at scheduled intervals led to increased and sustainable engraftment of microbiota from lean donors in obese recipients with T2DM that persisted for at least 6months (Ng et al., 2022). Mocanu et al. Also found that engraftment of specific taxa in the FMT plus low-fermentable fiber group at 6 weeks was donor mediated, and the FMT serves as fiber degrader, as well as short-chain fatty acid (SCFA)-producers and suppressors of tumor growth (Vindigni and Surawicz, 2017). Our FMT procedure led to a quick clinical response within 4 weeks and we found Bifidobacterium was successfully reconstituted in FMT treatment patients. Bifidobacterium plays an important role in human health, including regulating gut microbiota homeostasis, regulating local and systemic immune responses, inhibiting pathogens and harmful bacteria colonizing or infecting gut mucosa (Al-Salami et al., 2008), improving gut mucosal barrier and reducing gut lipopolysaccharide level (Ren et al., 2017). In addition, *Bifidobacteria* improves mucosal barrier function, and T2DM patients’ intake of probiotic yogurt containing Lactobacillus acidophilus La5 and Bifidobacterium lactis Bb12 for 6 weeks improved FBG and HbA1c (He et al., 2021).

Side effects of FMT include mild and self-limiting abdominal discomfort, cramps, abdominal distension, diarrhea or constipation, and very few diseases that cannot pass screening tests (Lu and Salzberg, 2020). In our study, T2DM patients displayed no adverse reactions after FMT, and the fasting and postprandial blood glucose, HbA1c, and insulin resistance decreased significantly without causing hypoglycemia or dyslipidemia after FMT treatment. In terms of metabolic research, previous studies tended to use FMT to intervene in metabolic syndrome, while few studies used FMT to intervene in T2DM. Our research is innovative. In a previous animal experiment, it was found that probiotics consumption in diabetes rats can increase the bioavailability of gliclazide (an oral sulfonylurea anti diabetes drug) (Segata et al., 2011). Our research found that FMT combined with metformin is better than metformin alone to achieve the improvement of blood glucose control and insulin resistance, which provides a new direction for FMT to intervene in T2DM and FMT combined with hypoglycemic drugs to intervene in T2DM.

Our study has several limitations. First, the relatively small sample size was not sufficient to evaluate the subtle differences or mechanisms associated with metformin treatment with or without FMT therapies. Second, the study period was limited to 4 weeks, restricting our understanding of the relationship between long-term clinical efficacy and engraftment of donor-associated microbiota. Third, due to scarcity of the donors, we used multi-donor FMT at different FMT times to enhance the microbial diversity transferred to recipients. However, a fixed donor choice based on donor-recipients matching for patients would help eliminate any confounding factors.

## 5 Conclusion

In conclusion, In conclusion, our study showed that FMT improved the BMI, PBG, HbA1c, FBG, HOMA-HBCI, and HOMA-IR of T2DM patients in 4 weeks and also promoted the engraftment of donor-associated microbiota in participants. Results from our trial will serve as a basis for the long-term intervention of FMT in T2DM patients and the further development of novel biotherapeutic strategies aimed at combatting T2DM through the safe, effective, and affordable bacterial formulations.

## Data availability statement

The datasets presented in this study can be found in online repositories. The names of the repository/repositories and accession number(s) can be found in the article/**Supplementary Material**.

## Ethics statement

The studies involving human participants were reviewed and approved by Longhu Hospital, The First Affiliated Hospital of Shantou University Medical College Ethics Committee in Shantou, China(Ethics number:LHLL2019001), and was registered at Chinses Clinical Trial Registry. (Registration number: ChiCTR1900024636).(http://www.chictr.org.cn/showprojen.aspx?proj=41166), and written informed consent was provided from all the patients. Written informed consent to participate in this study was provided by the participants’ legal guardian/next of kin.

## Author contributions

ZW and BZ, conceptualization, investigation, methodology, and writing-original draft. RX and FC, conceptualization, investigation, methodology, writing-review and editing. DZ and BC, conceptualization, investigation, writing-review and editing. AL and CZ, conceptualization, supervision, writing-review and editing. DH, XL, and SZ, conceptualization, investigation, writing-review and editing. KH and YC, conceptualization, formal analysis, investigation, visualization, supervision, writing-review and editing, project administration, and funding acquisition. All authors contributed to the article and approved the submitted version.

## Supplementary material

Supplementary Table 1 Basic information and clinical data of participants.

## References

[B1] Aggarwala V. Mogno I. Li Z. Yang C. Britton G. J. Chen-Liaw A.. (2021). Precise quantification of bacterial strains after fecal microbiota transplantation delineates long-term engraftment and explains outcomes. Nat. Microbiol. 6 (10), 1309–1318. doi: 10.1038/s41564-021-00966-0 34580445 PMC8993687

[B2] Aguayo-Mazzucato C. Andle J. Lee T. B. Jr. Midha A. Talemal L. Chipashvili V.. (2019). Acceleration of beta cell aging determines diabetes and senolysis improves disease outcomes. Cell Metab. 30 (1), 129–42.e4. doi: 10.1016/j.cmet.2019.05.006 31155496 PMC6610720

[B3] Al-Salami H. Butt G. Fawcett J. P. Tucker I. G. Golocorbin-Kon S. Mikov M. (2008). Probiotic treatment reduces blood glucose levels and increases systemic absorption of gliclazide in diabetic rats. Eur. J. Drug Metab. Pharmacokinet. 33 (2), 101–106. doi: 10.1007/BF03191026 18777945

[B4] Aron-Wisnewsky J. Clement K. Nieuwdorp M. (2019). Fecal microbiota transplantation: a future therapeutic option for Obesity/Diabetes? Curr. Diabetes Rep. 19 (8), 51. doi: 10.1007/s11892-019-1180-z 31250122

[B5] Belenguer A. Duncan S. H. Calder A. G. Holtrop G. Louis P. Lobley G. E.. (2006). Two routes of metabolic cross-feeding between bifidobacterium adolescentis and butyrate-producing anaerobes from the human gut. Appl. Environ. Microbiol. 72 (5), 3593–3599. doi: 10.1128/AEM.72.5.3593-3599.2006 16672507 PMC1472403

[B6] Bordalo Tonucci L. Dos Santos K. M. De Luces Fortes Ferreira C. L. Ribeiro S. M. De Oliveira L. L. Martino H. S. (2017). Gut microbiota and probiotics: Focus on diabetes mellitus. Crit. Rev. Food Sci. Nutr. 57 (11), 2296–2309. doi: 10.1080/10408398.2014.934438 26499995

[B7] Buse J. B. DeFronzo R. A. Rosenstock J. Kim T. Burns C. Skare S.. (2016). The primary glucose-lowering effect of metformin resides in the gut, not the circulation: Results from short-term pharmacokinetic and 12-week dose-ranging studies. Diabetes Care 39 (2), 198–205. doi: 10.2337/dc15-0488 26285584

[B8] Carvalho B. M. Saad M. J. (2013). Influence of gut microbiota on subclinical inflammation and insulin resistance. Mediators Inflamm 2013, 986734. doi: 10.1155/2013/986734 23840101 PMC3694527

[B9] Chen P. C. Chien Y. W. Yang S. C. (2019). The alteration of gut microbiota in newly diagnosed type 2 diabetic patients. Nutr. (Burbank Los Angeles County Calif) 63-64, 51–56. doi: 10.1016/j.nut.2018.11.019 30933725

[B10] Chudnovskiy A. Mortha A. Kana V. Kennard A. Ramirez J. D. Rahman A.. (2016). Host-protozoan interactions protect from mucosal infections through activation of the inflammasome. Cell 167 (2), 444–456.e14. doi: 10.1016/j.cell.2016.08.076 27716507 PMC5129837

[B11] DeFronzo R. A. Buse J. B. Kim T. Burns C. Skare S. Baron A.. (2016). Once-daily delayed-release metformin lowers plasma glucose and enhances fasting and postprandial GLP-1 and PYY: results from two randomised trials. Diabetologia 59 (8), 1645–1654. doi: 10.1007/s00125-016-3992-6 27216492 PMC4930485

[B12] de Groot P. Nikolic T. Pellegrini S. Sordi V. Imangaliyev S. Rampanelli E.. (2021). Faecal microbiota transplantation halts progression of human new-onset type 1 diabetes in a randomised controlled trial. Gut 70 (1), 92–105. doi: 10.1136/gutjnl-2020-322630 33106354 PMC7788262

[B13] de la Cuesta-Zuluaga J. Mueller N. T. Corrales-Agudelo V. Velasquez-Mejia E. P. Carmona J. A. Abad J. M.. (2017). Metformin is associated with higher relative abundance of mucin-degrading akkermansia muciniphila and several short-chain fatty acid-producing microbiota in the gut. Diabetes Care 40 (1), 54–62. doi: 10.2337/dc16-1324 27999002

[B14] Depommier C. Van Hul M. Everard A. Delzenne N. M. De Vos W. M. Cani P. D. (2020). Pasteurized akkermansia muciniphila increases whole-body energy expenditure and fecal energy excretion in diet-induced obese mice. Gut Microbes 11 (5), 1231–1245. doi: 10.1080/19490976.2020.1737307 32167023 PMC7524283

[B15] Duparc T. Plovier H. Marrachelli V. G. Van Hul M. Essaghir A. Stahlman M.. (2017). Hepatocyte MyD88 affects bile acids, gut microbiota and metabolome contributing to regulate glucose and lipid metabolism. Gut 66 (4), 620–632. doi: 10.1136/gutjnl-2015-310904 27196572 PMC5529962

[B16] Erem C. Ozbas H. M. Nuhoglu I. Deger O. Civan N. Ersoz H. O. (2014). Comparison of effects of gliclazide, metformin and pioglitazone monotherapies on glycemic control and cardiovascular risk factors in patients with newly diagnosed uncontrolled type 2 diabetes mellitus. Exp. Clin. Endocrinol. Diabetes 122 (5), 295–302. doi: 10.1055/s-0034-1370989 24710641

[B17] Foretz M. Guigas B. Viollet B. (2019). Understanding the glucoregulatory mechanisms of metformin in type 2 diabetes mellitus. Nat. Rev. Endocrinol. 15 (10), 569–589. doi: 10.1038/s41574-019-0242-2 31439934

[B18] Giongo A. Gano K. A. Crabb D. B. Mukherjee N. Novelo L. L. Casella G.. (2011). Toward defining the autoimmune microbiome for type 1 diabetes. ISME J. 5 (1), 82–91. doi: 10.1038/ismej.2010.92 20613793 PMC3105672

[B19] Hartstra A. V. Bouter K. E. Backhed F. Nieuwdorp M. (2015). Insights into the role of the microbiome in obesity and type 2 diabetes. Diabetes Care 38 (1), 159–165. doi: 10.2337/dc14-0769 25538312

[B20] He J. He X. Ma Y. Yang L. Fang H. Shang S.. (2021). A comprehensive approach to stool donor screening for faecal microbiota transplantation in China. Microb. Cell Fact. 20 (1), 216. doi: 10.1186/s12934-021-01705-0 34838016 PMC8626716

[B21] Herfarth H. Barnes E. L. Long M. D. Isaacs K. L. Leith T. Silverstein M.. (2019). Combined endoscopic and oral fecal microbiota transplantation in patients with antibiotic-dependent pouchitis: Low clinical efficacy due to low donor microbial engraftment. Inflamm. Gut. Dis. 4 (1), 1–6. doi: 10.1159/000497042 PMC6537468 31172007

[B22] Hou K. Wu Z. X. Chen X. Y. Wang J. Q. Zhang D. Xiao C.. (2022). Microbiota in health and diseases. Signal Transduct. Target. Ther. 7 (1), 135. doi: 10.1038/s41392-022-00974-4 35461318 PMC9034083

[B23] Hou K. Zhang S. Wu Z. Zhu D. Chen F. Lei Z. N.. (2022). Reconstruction of gut microecology of type 2 diabetes by fecal microbiota transplantation: Why and how. Bosnian J. Basic Med. Sci. 22 (3), 315–325. doi: 10.17305/bjbms.2021.6323 PMC9162745 34761734

[B24] Ingle H. Lee S. Ai T. Orvedahl A. Rodgers R. Zhao G.. (2019). Viral complementation of immunodeficiency confers protection against enteric pathogens *via* interferon-lambda. Nat. Microbiol. 4 (7), 1120–1128. doi: 10.1038/s41564-019-0416-7 30936486 PMC6588490

[B25] Karlsson F. H. Tremaroli V. Nookaew I. Bergstrom G. Behre C. J. Fagerberg B.. (2013). Gut metagenome in European women with normal, impaired and diabetic glucose control. Nature 498 (7452), 99–103. doi: 10.1038/nature12198 23719380

[B26] Kootte R. S. Levin E. Salojarvi J. Smits L. P. Hartstra A. V. Udayappan S. D.. (2017). Improvement of insulin sensitivity after lean donor feces in metabolic syndrome is driven by baseline gut microbiota composition. Cell Metab. 26 (4), 611–9.e6. doi: 10.1016/j.cmet.2017.09.008 28978426

[B27] Krentz A. J. Bailey C. J. (2005). Oral antidiabetic agents: current role in type 2 diabetes mellitus. Drugs 65 (3), 385–411. doi: 10.2165/00003495-200565030-00005 15669880

[B28] Lee P. Yacyshyn B. R. Yacyshyn M. B. (2019). Gut microbiota and obesity: An opportunity to alter obesity through faecal microbiota transplant (FMT). Diabetes Obes. Metab. 21 (3), 479–490. doi: 10.1111/dom.13561 30328245

[B29] Li X. V. Leonardi I. Iliev I. D. (2019). Gut mycobiota in immunity and inflammatory disease. Immunity 50 (6), 1365–1379. doi: 10.1016/j.immuni.2019.05.023 31216461 PMC6585451

[B30] Li X. Watanabe K. Kimura I. (2017). Gut microbiota dysbiosis drives and implies novel therapeutic strategies for diabetes mellitus and related metabolic diseases. Front. Immunol. 8, 1882. doi: 10.3389/fimmu.2017.01882 29326727 PMC5742320

[B31] Li S. S. Zhu A. Benes V. Costea P. I. Hercog R. Hildebrand F.. (2016). Durable coexistence of donor and recipient strains after fecal microbiota transplantation. Sci. (New York NY) 352 (6285), 586–589. doi: 10.1126/science.aad8852 27126044

[B32] Lu J. Salzberg S. L. (2020). Ultrafast and accurate 16S rRNA microbial community analysis using kraken 2. Microbiome 8 (1), 124. doi: 10.1186/s40168-020-00900-2 32859275 PMC7455996

[B33] Ma Q. Li Y. Li P. Wang M. Wang J. Tang Z.. (2019). Research progress in the relationship between type 2 diabetes mellitus and gut microbiota. Biomed. pharmacother. = Biomed. pharmacother. 117, 109138. doi: 10.1016/j.biopha.2019.109138 31247468

[B34] Marchesi J. R. Adams D. H. Fava F. Hermes G. D. Hirschfield G. M. Hold G.. (2016). The gut microbiota and host health: a new clinical frontier. Gut 65 (2), 330–339. doi: 10.1136/gutjnl-2015-309990 26338727 PMC4752653

[B35] Mayo B. Sinderen D. V. (2010). Bifidobacteria genomics and molecular aspects.

[B36] Mocanu V. Zhang Z. Deehan E. C. Kao D. H. Hotte N. Karmali S.. (2021). Fecal microbial transplantation and fiber supplementation in patients with severe obesity and metabolic syndrome: a randomized double-blind, placebo-controlled phase 2 trial. Nat. Med. 27 (7), 1272–1279. doi: 10.1038/s41591-021-01399-2 34226737

[B37] Musso G. Gambino R. Cassader M. (2011). Interactions between gut microbiota and host metabolism predisposing to obesity and diabetes. Annu. Rev. Med. 62, 361–380. doi: 10.1146/annurev-med-012510-175505 21226616

[B38] Ng S. C. Xu Z. Mak J. W. Y. Yang K. Liu Q. Zuo T.. (2022). Microbiota engraftment after faecal microbiota transplantation in obese subjects with type 2 diabetes: a 24-week, double-blind, randomised controlled trial. Gut 71 (4), 716–723. doi: 10.1136/gutjnl-2020-323617 33785557

[B39] Pinzone M. R. Celesia B. M. Di Rosa M. Cacopardo B. Nunnari G. (2012). Microbial translocation in chronic liver diseases. Int. J. Microbiol. 2012, 694629. doi: 10.1155/2012/694629 22848224 PMC3405644

[B40] Pollak M. (2017). The effects of metformin on gut microbiota and the immune system as research frontiers. Diabetologia 60 (9), 1662–1667. doi: 10.1007/s00125-017-4352-x 28770326

[B41] Que Y. Cao M. He J. Zhang Q. Chen Q. Yan C.. (2021). Gut bacterial characteristics of patients with type 2 diabetes mellitus and the application potential. Front. Immunol. 12, 722206. doi: 10.3389/fimmu.2021.722206 34484230 PMC8415158

[B42] Ren Y. D. Ye Z. S. Yang L. Z. Jin L. X. Wei W. J. Deng Y. Y.. (2017). Fecal microbiota transplantation induces hepatitis b virus e-antigen (HBeAg) clearance in patients with positive HBeAg after long-term antiviral therapy. Hepatol. (Baltimore Md) 65 (5), 1765–1768. doi: 10.1002/hep.29008 28027582

[B43] Rinott E. Youngster I. Yaskolka Meir A. Tsaban G. Zelicha H. Kaplan A.. (2021). Effects of diet-modulated autologous fecal microbiota transplantation on weight regain. Gastroenterology 160 (1), 158–73.e10. doi: 10.1053/j.gastro.2020.08.041 32860791 PMC7755729

[B44] Rodriguez J. Hiel S. Delzenne N. M. (2018). Metformin: old friend, new ways of action-implication of the gut microbiome? Curr. Opin. Clin. Nutr. Metab. Care 21 (4), 294–301. doi: 10.1097/MCO.0000000000000468 29634493

[B45] Segata N. Izard J. Waldron L. Gevers D. Miropolsky L. Garrett W. S.. (2011). Metagenomic biomarker discovery and explanation. Genome Biol. 12 (6), R60. doi: 10.1186/gb-2011-12-6-r60 21702898 PMC3218848

[B46] Shin N. R. Lee J. C. Lee H. Y. Kim M. S. Whon T. W. Lee M. S.. (2014). An increase in the akkermansia spp. population induced by metformin treatment improves glucose homeostasis in diet-induced obese mice. Gut 63 (5), 727–735. doi: 10.1136/gutjnl-2012-303839 23804561

[B47] Skelly A. N. Sato Y. Kearney S. Honda K. (2019). Mining the microbiota for microbial and metabolite-based immunotherapies. Nat. Rev. Immunol. 19 (5), 305–323. doi: 10.1038/s41577-019-0144-5 30858494

[B48] Su L. Hong Z. Zhou T. Jian Y. Xu M. Zhang X.. (2022). Health improvements of type 2 diabetic patients through diet and diet plus fecal microbiota transplantation. Sci. Rep. 12 (1), 1152. doi: 10.1038/s41598-022-05127-9 35064189 PMC8782834

[B49] Su B. Liu H. Li J. Sunli Y. Liu B. Liu D.. (2015). Acarbose treatment affects the serum levels of inflammatory cytokines and the gut content of bifidobacteria in Chinese patients with type 2 diabetes mellitus. J. Diabetes 7 (5), 729–739. doi: 10.1111/1753-0407.12232 25327485

[B50] Sun L. Xie C. Wang G. Wu Y. Wu Q. Wang X.. (2018). Gut microbiota and gut FXR mediate the clinical benefits of metformin. Nat. Med. 24 (12), 1919–1929. doi: 10.1038/s41591-018-0222-4 30397356 PMC6479226

[B51] Thingholm L. B. Ruhlemann M. C. Koch M. Fuqua B. Laucke G. Boehm R.. (2019). Obese individuals with and without type 2 diabetes show different gut microbial functional capacity and composition. Cell Host Microbe 26 (2), 252–64.e10. doi: 10.1016/j.chom.2019.07.004 31399369 PMC7720933

[B52] Tolhurst G. Heffron H. Lam Y. S. Parker H. E. Habib A. M. Diakogiannaki E.. (2012). Short-chain fatty acids stimulate glucagon-like peptide-1 secretion *via* the G-protein-coupled receptor FFAR2. Diabetes 61 (2), 364–371. doi: 10.2337/db11-1019 22190648 PMC3266401

[B53] Turnbaugh P. J. Hamady M. Yatsunenko T. Cantarel B. L. Duncan A. Ley R. E.. (2009). A core gut microbiome in obese and lean twins. Nature 457 (7228), 480–484. doi: 10.1038/nature07540 19043404 PMC2677729

[B54] Vindigni S. M. Surawicz C. M. (2017). Fecal microbiota transplantation. Gastroenterol. Clinics North Am 46 (1), 171–185. doi: 10.1016/j.gtc.2016.09.012 28164849

[B55] Vrieze A. Van Nood E. Holleman F. Salojarvi J. Kootte R. S. Bartelsman J. F.. (2012). Transfer of gut microbiota from lean donors increases insulin sensitivity in individuals with metabolic syndrome. Gastroenterology 143 (4), 913–6.e7. doi: 10.1053/j.gastro.2012.06.031 22728514

[B56] Wallace H. J. Holmes L. Ennis C. N. Cardwell C. R. Woodside J. V. Young I. S.. (2019). Effect of vitamin D3 supplementation on insulin resistance and beta-cell function in prediabetes: a double-blind, randomized, placebo-controlled trial. Am. J. Clin. Nutr. 110 (5), 1138–1147. doi: 10.1093/ajcn/nqz171 31559433

[B57] Wang H. Lu Y. Yan Y. Tian S. Zheng D. Leng D.. (2019). Promising treatment for type 2 diabetes: Fecal microbiota transplantation reverses insulin resistance and impaired islets. Front. Cell. infect. Microbiol. 9, 455. doi: 10.3389/fcimb.2019.00455 32010641 PMC6979041

[B58] Wild S. Roglic G. Green A. Sicree R. King H. (2004). Global prevalence of diabetes: estimates for the year 2000 and projections for 2030. Diabetes Care 27 (5), 1047–1053. doi: 10.2337/diacare.27.5.1047 15111519

[B59] Wu G. D. Chen J. Hoffmann C. Bittinger K. Chen Y. Y. Keilbaugh S. A.. (2011). Linking long-term dietary patterns with gut microbial enterotypes. Sci. (New York NY) 334 (6052), 105–108. doi: 10.1126/science.1208344 PMC3368382 21885731

[B60] Wu Z. Chen Y. Zhu D. Zheng Y. Ali K. B. Hou K. (2022). Advancement of traditional Chinese medicine in regulation of gut microbiota: Mechanism-based role in disease management. Recent patents anti-cancer Drug Discovery 17 (2), 136–144. doi: 10.2174/1574892816666210929164930 34587887

[B61] Wu H. Esteve E. Tremaroli V. Khan M. T. Caesar R. Manneras-Holm L.. (2017). Metformin alters the gut microbiome of individuals with treatment-naive type 2 diabetes, contributing to the therapeutic effects of the drug. Nat. Med. 23 (7), 850–858. doi: 10.1038/nm.4345 28530702

[B62] Wu H. Tremaroli V. Schmidt C. Lundqvist A. Olsson L. M. Kramer M.. (2020). The gut microbiota in prediabetes and diabetes: A population-based cross-sectional study. Cell Metab. 32 (3), 379–90.e3. doi: 10.1016/j.cmet.2020.06.011 32652044

[B63] Yadav H. Lee J. H. Lloyd J. Walter P. Rane S. G. (2013). Beneficial metabolic effects of a probiotic *via* butyrate-induced GLP-1 hormone secretion. J. Biol. Chem. 288 (35), 25088–25097. doi: 10.1074/jbc.M113.452516 23836895 PMC3757173

[B64] Zhao L. Zhang F. Ding X. Wu G. Lam Y. Y. Wang X.. (2018). Gut bacteria selectively promoted by dietary fibers alleviate type 2 diabetes. Sci. (New York NY) 359 (6380), 1151–1156. doi: 10.1126/science.aao5774 29590046

